# Utility of direct 3D co-culture model for chondrogenic differentiation of mesenchymal stem cells on hyaluronan scaffold (Hyaff-11)

**DOI:** 10.1093/rb/rbaa026

**Published:** 2020-08-23

**Authors:** Iwona Deszcz, Anna Lis-Nawara, Piotr Grelewski, Szymon Dragan, Julia Bar

**Affiliations:** 1 Department of Immunopathology and Molecular Biology, Wroclaw Medical University, Bujwida 44, 50-345 Wroclaw, Poland; 2 Department and Clinic of Orthopedic and Traumatologic Surgery, Wroclaw Medical University, Bujwida 44, 50-345 Wroclaw, Poland

**Keywords:** articular cartilage regeneration, chondrogenesis, direct co-culture, Hyaff-11

## Abstract

This study presents direct 2D and 3D co-culture model of mesenchymal stem cells (MSCs) line with chondrocytes isolated from patients with osteoarthritis (unaffected area). MSCs differentiation into chondrocytes after 14, 17 days was checked by estimation of collagen I, II, X, aggrecan expression using immunohistochemistry. Visualization, localization of cells on Hyaff-11 was performed using enzymatic technique and THUNDER Imaging Systems. Results showed, that MSCs/chondrocytes 3D co-culture induced suitable conditions for chondrocytes grow and MSCs differentiation than 2D monoculture. Despite that differentiated cells on Hyaff-11 expressed collagen X, they showed high collagen II (80%) and aggrecan (60%) expression with simultaneous decrease of collagen I expression (10%). The high concentration of differentiated cells on Hyaff-11, indicate that this structure has an impact on cells cooperation and communication. In conclusion, we suggest that high expression of collagen II and aggrecan in 3D co-culture model, indicate that cooperation between different subpopulations may have synergistic impact on MSCs chondrogenic potential. Revealed the high concentration and localization of cells growing in deeper layers of membrane in 3D co-culture, indicate that induced microenvironmental enhance cell migration within scaffold. Additionally, we suggest that co-culture model might be useful for construction a bioactive structure for cartilage tissue regeneration.

## Introduction

Degenerative diseases and mechanical injuries of the articular cartilage limit the motor skills of a large population, regardless of the age range. Currently, most of the clinical approach used to repair tissue defects within the articular cartilage include abrasion and microfracture, which are intended to stimulate cartilage growth, which should lead to its regeneration [1, 2]. It should be emphasized that in the majority of patients, the radical treatment of a damaged knee or hip-joint is replaced by an artificial endoprosthesis, which should be replaced after some time [3]. A breakthrough approach for the treatment of full-thickness cartilage occurred when Peterson and Brittberg [4] published a report describing the autologous chondrocyte implantation technique. This method has proven to be a promising treatment option to restore hyaline-like cartilage [5]. However, autologous chondrocyte transplantation has disadvantages, because chondrocytes undergo rapid dedifferentiation during *in vitro* expansion, resulting in decreasing or no presence of type II collagen and increasing level of type I and X collagen [6]. These changes may lead to the formation of fibrocartilage with inferior mechanical properties [6]. That is why improvements of this technique are still being sought. Cartilage tissue engineering involves the use of 3D structures on which the cells are seeded. However, there are some limitations of this approach: one of them is finding an ideal scaffold, the second is the source of stem cells and the third are factors which induce appropriate environmental [7, 8]. The ideal scaffold should degrade and resorb at a rate corresponding to tissue growth, thereby defining the shape and function of the assimilated tissue structure and should maintain the chondrogenic phenotype of the cells [9]. The most popular 3D structures are made of natural biodegradable materials such as collagen, fibrinogen or hyaluronic acid [10]. The collagen scaffold is commonly used in articular cartilage repair, because it provides suitable conditions for chondrocyte differentiation [7, 11]. Moreover, type II collagen and proteoglycans are main components of the articular cartilage extracellular matrix, which maintains the chondrocyte phenotype [10–13]. The second component of the articular cartilage is hyaluronic acid [13]. Autologous cell therapies use scaffold made of hyaluronic acid benzyl ester polymer (Hyaff-11), which supports chondrocytes growth and maintains their original phenotype. Clinical use of Hyaff-11 gave very good results [11, 14–17].

The ability of mesenchymal stem cells (MSCs) to self-renew has been identified as well as their ability to differentiate into several tissues. They appear to be an appropriate material for tissue engineering and regeneration [18]. Stem cells therapy is based on the injection of cells suspension or the introduction of cells seeded on a scaffold in place of the damaged tissue [7]. However, chondrogenic differentiation of MSCs shows the hypertrophic phenotype and the calcification of obtained chondrocytes [6]. Therefore, the new approach, based on a combination of MSCs with chondrocytes on scaffolds, has shown promising results [19–21]. The co-culture stimulates both populations of cells: chondrocytes used to promote chondrocyte differentiation of MSCs and MSCs generate instructive signals, increase chondrogenic capacity of chondrocytes, secrete a wide range of bioactive factors and matrix molecules like collagen, proteoglycans as well as cytokines and growth factors [20, 22, 23]. However, it was revealed that biological properties of 3D structures depend on the co-culture model used. The first model is a direct co-culture where MSCs are cultured together on the culture dish or a scaffold with chondrocytes; the second one is indirect co-culture, where MSCs are separated by a barrier (transmembrane) from chondrocytes [19]. Chondrogenesis of MSCs in direct co-culture is effective, because primary chondrocytes have the ability to gradually secrete a variety of protein molecules, like TGF-β, IGF-1, BMP-2 and FGF-2 [23]. This can better affect *in vivo* MSC-assisted cartilage regeneration in the absence of exogenous inducers and MSCs secret cytokines, growth factors, exhibiting anti-inflammatory effects [19, 23]. Direct cooperation between primary chondrocytes and MSCs revealed higher expression of SOX9, type II collagen, and aggrecan in chondrocytes compared with the standard model [24]. On the other hand, indirect co-culture probably could reduce the hypertrophic phenotype of both cell types [1]. Although both co-cultures, direct and indirect, have advantages, it is still debatable which system will be better in clinical trials. Growing evidence suggests that the cooperation between stem cells, membrane and grow factors has an impact on the biological function of 3D structure. Moreover, it is not clear which parameters are essential to design the appropriate bioactive construction, showing high differentiation potential of stem cells.

The aim of the study was to analyze and compare the expression of type I, II, X collagen and aggrecan depend on culture condition use to MSCs differentiation toward chondrocytes.

## Materials and methods

### Biological material

In this study, commercial cell line of MSCs, bone marrow (Provitro AG, Berlin, Germany) and chondrocytes isolated from knee cartilage of patients (human knee cartilage) was used. According to manufactures description MSCs line was positive for CD44, CD90, CD73, CD105, CD166 and negative for MAC-1, CD14, CD19, CD34, CD45, HLA-DR. Normal human knee cartilage (no more than 100 mg) was obtained from three patients (two women and one man with mean age of (57 ± 14) years) with osteoarthritis during arthroscopic regenerative procedures. The tissue was collected from unaffected area distal to the damaged zone. The chondrocytes isolation was performed according to earlier data [25]. Cells were cultured in Dulbecco’s modified Eagle’s medium (Gibco, Karlsruhe, Germany) with 10% fetal calf serum (Gibco, Karlsruhe, Germany) and supplemented with antibiotics (penicillin/streptomycin; Sigma-Aldrich, Poznań, Poland) in a humidified atmosphere at 37°C and 5% CO_2_. The Trypsin 0.25% (Sigma-Aldrich, Poznań, Poland) was used to remove cells from culture flasks for further experiments. For *in vitro* study, three cases were used. All *in vitro* experiments were repeated three times. Patients who participated in the study signed an informed consent, after detailed explanation of the study related issues. Ethical agreement for the research has been obtained (no. KB-502/2017). The cells were stained using hematoxylin–eosin staining for morphological assessment.

### Direct 2D and 3D co-culture

Co-culture was performed using MSCs and chondrocytes in cultures flasks (2D) and on hyaluronic acid membrane Hyaff-11 (HyaloFast; Anika Therapeutics, Padova, Italy) using supplemented Dulbecco’s modified Eagle’s medium for 14 and 17 days. The equal amount of MSCs and chondrocytes (1:1) were applied into culture flask or Hyaff-11. The medium was changed twice a week. The cells were stained using hematoxylin–eosin staining for morphological assessment.

### Chondrogenesis of MSCs in 2D and 3D culture

Chondrogenesis was performed using MSCs in cultures flasks (2D) and on Hyaff-11 (3D) using hMSC chondrogenesis induction medium (hMSC; Provitro AG, Berlin, Germany) for 14 and 17 days. The chondrogenic medium was changed twice a week. The cells were stained using hematoxylin–eosin staining for morphological assessment.

### Antibodies

For immunohistochemical staining the following monoclonal antibodies were used: CD44 (156-3C11, ThermoFisher Scientific, Rockford, IL, USA), CD90 (AF-9; ThermoFisher Scientific, Rockford, IL, USA), CD105 (MEM-229; ThermoFisher Scientific, Rockford, IL, USA), type I collagen (COL-1; ThermoFisher Scientific, Rockford, IL, USA), type II collagen (M2139; ThermoFisher Scientific, Rockford, IL, USA), type X collagen (Col-10; Sigma-Aldrich, St. Louis, MO, USA), vimentin (anti-vimentin clone V9; Dako, Copenhagen, Denmark) and aggrecan (catalog nr AHP0022 Anti-human Agrgecan; Invitrogen, Carslbad, CA, USA).

### Immunohistochemistry

Immunohistochemistry was performed on cells which were: (i) previously removed from culture flasks or from 3D membrane using the Trypsin 0.25% and fixed on slides; (ii) fixed on the membrane fibers in formaldehyde or ice cold acetone. Next, the Universal Dako REAL EnVision detection system, peroxidase/DAB+, rabbit/mouse (Dako, Copenhagen, Denmark) were used. Then, cell samples were incubated with primary antibodies (CD44, CD90, CD105, type I, II and X collagen, vimentin) for 1.5 h at room temperature and aggrecan, for 24 h at 4°C. The antigen–antibody reaction was visualized by DAB (3,3 diaminobenzidine) (Dako, Copenhagen, Denmark) as a chromogen for 4 min at room temperature and counterstained with hematoxylin and mounted. The samples incubated without primary antibodies were used as negative controls.

### Immunohistochemical staining interpretation

The percentage of immunopositive cells were determined by counting 700 cells in randomly selected areas, by two independent evaluators, using Olympus microscope BX51 (Olympus, Tokyo, Japan). The expression of CD44, CD90, CD105, type I and II, X collagen, vimentin and aggrecan proteins were assessed by determining the number of cells exhibiting a immunoreaction. Positive staining required more than 5% of cells showing reaction. The expression of analyzed markers in cells located on Hyaff-11 fibers was analyzed by estimating the intensity of immunostaining. The intensity score was based on the color of reaction, where 0 = no immunostaining, light yellow color = weak (+), medium brown color = moderate (++) and brown color = strong (+++).

### MTT assay: determination of cells viability

The non-cytotoxicity of Hyaff-11 has been confirmed by the survival of cells cultured on Hyaff-11 was determined by the MTT assay (in vitro toxicology assay; Sigma-Aldrich, Poznan Poland). This test checks the mitochondrial metabolic functioning (NADH activity) with the 3-(4,5-dimethylthiazol-2-yl)-2,5-diphenyltetrazolium bromide (MTT). The MSCs or chondrocytes growing on membrane for 48 h were transferred to 96-well plate and incubate at 37°C in a 5% CO_2_ overnight. Next, the cells were treated according to the manufacturer’s protocol. The absorbance at 570 nm was measured using multiwall scanning spectrophotometer (EnSpire Multimode Reader; Perkin Elmer, Kraków, Poland). The results were expressed as a percentage of mitochondrial function relative to cells growing 2D.

### The cells viability: trypan blue staining

To confirm the viability of cells growing on the Hyaff-11, trypan blue (Trypan Blue solution, Sigma-Aldrich, Poznan Poland) was used. To slightly highlight the cells located on the membrane’s fibers and to loosen the connections between fibers and cells, trypsin for 1 min was used (PL Patent Application number P.425169-owners J.B., I.D., S.D.). Next, the mixture of PBS and trypan blue was added to a petri dish, and under light microscopy the cells were analyzed (viable cells have a clear cytoplasm, dead cells have a blue color).

### Estimation of cells localization on 3D membrane

To check the cells localization on membrane, we used the method protected by our PL Patent Application number P.425169 and THUNDER Imaging Systems (Leica, Zalesie Gorne, Poland). Collagens present on the membrane and single fibers was observed using THUNDER Imaging Systems at wave length 470 nm.

### Statistical analysis

All quantitative data are expressed as mean ± standard deviation and differences between mean values were analyzed using ANOVA test. A *P* value ≤0.05 was considered statistically significant.

## Results

### Immunophenotype of chondrocytes growing in 2D and 3D culture

Chondrocytes cultured on 2D revealed differences in the type I and II collagen expression, which depend on the time of culturing. The decrease of type II collagen and increase type I collagen was observed starting from passage 2 and finally achieved 0 and 80%, respectively, at passage 3 (Table 1). There was significant decrease of aggrecan expression in chondrocytes from 60% immunopositive cells at passage 1–20% at passage 3 (*P *=* *0.03). The presence of type X collagen was observed in cells at passage 1 and did not changed significantly during culturing. Chondrocytes with low expression of type II collagen growing on 3D structure showed an increased level of this protein to 70% (3D versus passage 1 (*P *=* *0.009); versus passage 2 (*P *=* *0.001); versus passage 3 (*P *=* *0.0008)) and a decreased level of type I collagen expression to 20% (3D versus passage 2 (*P *=* *0.03); versus passage 3 (*P *=* *0.03)). The significant differences were observed between type X collagen expression in chondrocytes cultured 3D versus chondrocytes cultured 2D (passage 1 (*P *=* *0.0002); versus passage 2 (*P* = 0.00005); versus passage 3 (*P *=* *0.0001)).

**Table 1:** Immunophenotype of chondrocytes growing in 2D and 3D culture

	2D culture			3D culture	
Biomarkers	Passage			Cells removed from scaffold	Cells located on scaffold
	1	2	3	14 days	
	Immunoreactivity (% of positive cells)			Immunoreactivity (% of positive cells)	Intensity of immunostaining
Collagen I	40 ± 23	80 ± 6	80 ± 0	20 ± 12	+/++
Collagen II	15 ± 17	10 ± 6	0 ± 6	70 ± 13	+++
Collagen X	25 ± 6	20 ± 6	25 ± 6	80 ± 4	++/+++
Aggrecan	60 ± 10	40 ± 30	20 ± 10	40 ± 0	+/+++

### Chondrogenesis of MSCs in 2D culture

The ability of MSCs to differentiate into chondrocytes was analyzed with reference to the expression of type I, II, X collagen and aggrecan in cells after 14 and 17 days of cultivation. It was found that the expression of type I collagen clearly decreased after 17 days of culture compared with 14 days from 60 to 20% of positive cells (*P *=* *0.000001) and decrease of type X collagen after 17 days of culture (*P *=* *0.01) (Table 2). No type II collagen expression was observed at the analyzed time points, but aggrecan started to be visible after 17 days of culture (*P *=* *0.004). Heterogeneous patterns of type I collagen and aggrecan immunostaining were observed in cells after differentiation. The stem cells markers (such as CD44, CD90, CD105) expression was lower in cells after 17 days of differentiation (*P *=* *0.0000) (Table 2).

**Table 2:** Comparison of biomarkers expression in chondroinduced MSCs and MSCs/chondrocytes in direct co-culture in 2D and 3D model

Biomarkers	Chondroinduced MSCs				Direct co-culture			
	14 days		17 days		14 days		17 days	
	2D	3D	2D	3D	2D	3D	2D	3D
	Immunoreactivity (% of positive cells)							
CD44	100 ± 3	80 ± 3	80 ± 0	90 ± 0	90 ± 3	80 ± 5	95 ± 3	90 ± 3
CD90	65 ± 5	50 ± 0	30 ± 3	90 ± 0	80 ± 3	0 ± 3	80 ± 4	95 ± 4
CD105	50 ± 3	90 ± 3	40 ± 3	80 ± 3	100 ± 3	60 ± 8	95 ± 3	95 ± 0
Collagen I	60 ± 3	40 ± 3	20 ± 3	55 ± 4	20 ± 3	40 ± 3	40 ± 3	10 ± 3
Collagen II	0 ± 0	50 ± 4	0 ± 0	80 ± 3	0 ± 3	10 ± 3	0 ± 3	80 ± 3
Collagen X	70 ± 4	90 ± 0	60 ± 3	80 ± 4	75 ± 6	90 ± 3	90 ± 3	80 ± 3
Aggrecan	0 ± 0	20 ± 3	10 ± 3	20 ± 0	80 ± 5	60 ± 6	40 ± 4	60 ± 4

### MSCs/chondrocytes in direct 2D co-culture system

**Figure 1:**
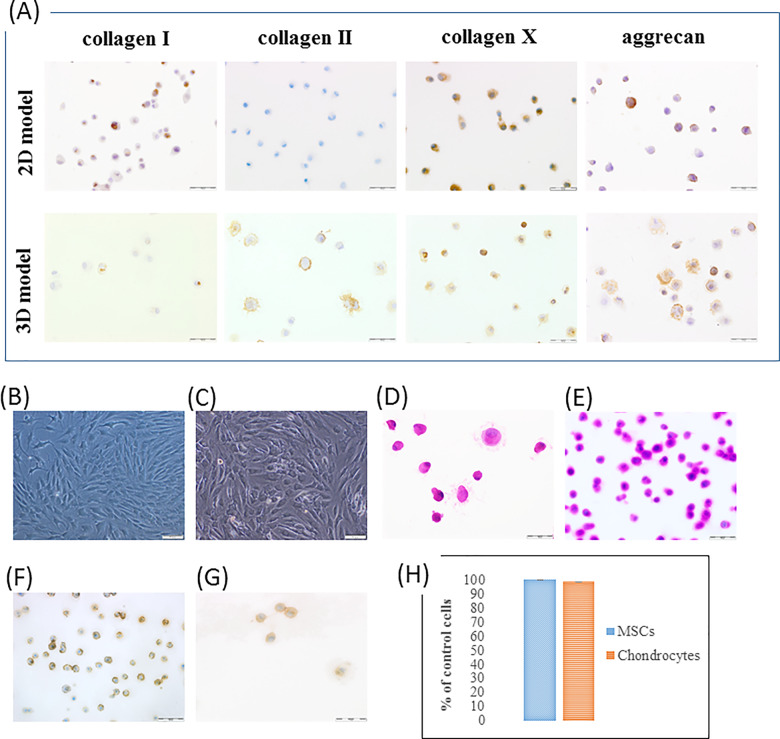
Immunophenotypic and morphological features of MSCs and chondrocytes in direct co-culture. (A) Immunohistochemical staining of type I, II, X collagen and aggrecan in MSCs/chondrocytes after 17 days of differentiation in direct 2D and 3D co-culture model (EnVision technique, scale bar = 50 µm); (B) Chondrocytes and (G) direct co-culture on culture flask (inverted phase contrast microscope, scale bar = 100 µm); (C) Hematoxylin–eosin staining of chondroinduced MSCs and (D) co-culture in 2D culture after 14 days (scale bar = 50 µm); (E) Expression of vimentin in MSCs/chondrocytes and (F) chondroinduced MSCs after 17 days of culture in direct 2D co-culture model (EnVision technique, scale bar = 50 µm); (H) The viability of MSCs and chondrocytes on the Hyaff-11 membrane after 48 h. Data are given as mean ± standard deviation.

Direct co-culture of MSCs with chondrocytes after 14 and 17 days of culture revealed that expression of type I and X collagen increased at the 17th day (*P *=* *0.0000; *P *=* *0.00002, respectively), whereas aggrecan decreased from 80 to 40% of positive cells (*P *=* *0.0000). We did not observe expression of type II collagen in both time points (Table 2) (Fig. 1A). The stem cells markers (such as CD44, CD90, CD105) presence revealed slight differences between time of differentiation (*P *>* *0.05).

### Comparison of analyzed biomarkers between chondroinduced MSCs and MSCs/chondrocytes in direct co-culture (2D models)

The differences between analyzed biomarkers were observed between the groups and corresponding time points. Significant differences were observed for CD44, CD90, CD105, type I collagen and aggrecan expression in chondroinduced MSCs after 14 days of culture versus direct co-culture model after 14 days of culture and versus 17 days of culture (*P *≤* *0.05) (Table 2). Type X collagen showed significant differences between chondroinduced MSCs after 14 days of culture versus direct co-culture model after 17 days of culture (*P *≤* *0.05). The expression of type I, X collagen and aggrecan in chondrocytes isolated from cartilage tissue showed differences in comparison versus chondroinduced MSCs (*P *=* *0.0000) and versus co-culture (*P *=* *0.0000; *P *=* *0.0000; *P *=* *0.00001 at 14th day, respectively) (Table 2).

### Chondrogenesis of MSCs on 3D membrane

**Figure 2:**
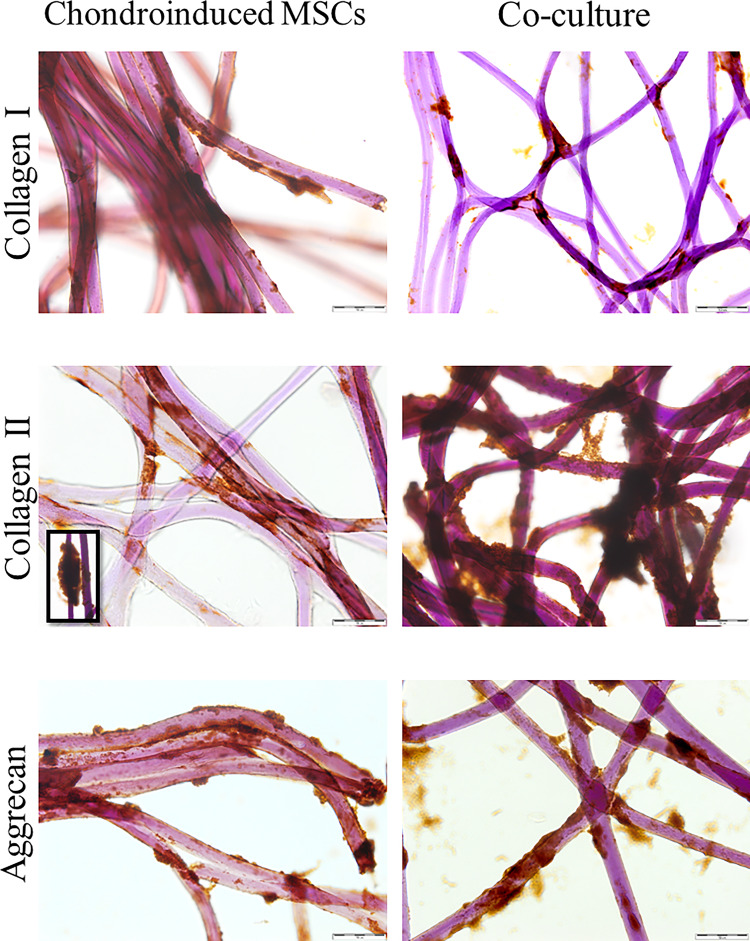
Immunohistochemical staining of type I, II collagen and aggrecan in MSCs in standard culture and MSCs/chondrocytes in direct co-culture model after 17 days differentiation on Hyaff-11 membrane (EnVision technique, scale bar = 50 µm, co-culture collagen I scale bar = 100 µm).

After 14 days of MSCs differentiation on 3D structure the expression of type I, II, X collagen and aggrecan was analyzed on fibers of the membrane (Fig. 2). Strong expression of aggrecan was observed (+++), whereas type I, II and X collagen expression revealed immunostaining from + to ++ (Table 3). In the next step of our experiment, chondroinduced MSCs were removed from the scaffold. The cells specimens showed increasing level of type I and II collagen expression after 17 days of culture compared with 14 days (*P *=* *0.0000; *P *=* *0.0000, respectively) (Table 2). The aggrecan expression was constant for both time points (20%; *P *>* *0.05) (Fig. 1A). The decrease of type X collagen was observed after 17 days of culture (*P *=* *0.00). The differences in stem cells markers (such as CD44, CD90, CD105) expression in cells after 17 days of differentiation in comparison to 14 day were significant (*P *=* *0.0000).

**Table 3:** Expression of analyzed markers on differentiated cells located on Hyaff-11 fibers (3D) after 14 and 17 days of culture

Biomarkers	Chondroinduced MSCs		Direct co-culture	
	14 days	17 days	14 days	17 days
	(Intensity of immunostaining)			
CD44	+/++	++/+++	++	+/++
CD90	++	++/+++	++	++
CD105	+	+/++	++	+/+++
Collagen I	+	+	+++	+/++
Collagen II	++/+++	++	+	+/++
Collagen X	++	−	+++	+/++
Aggrecan	+++	++	+/++	+/++

### MSCs/chondrocytes in direct 3D co-culture system

**Figure 3:**
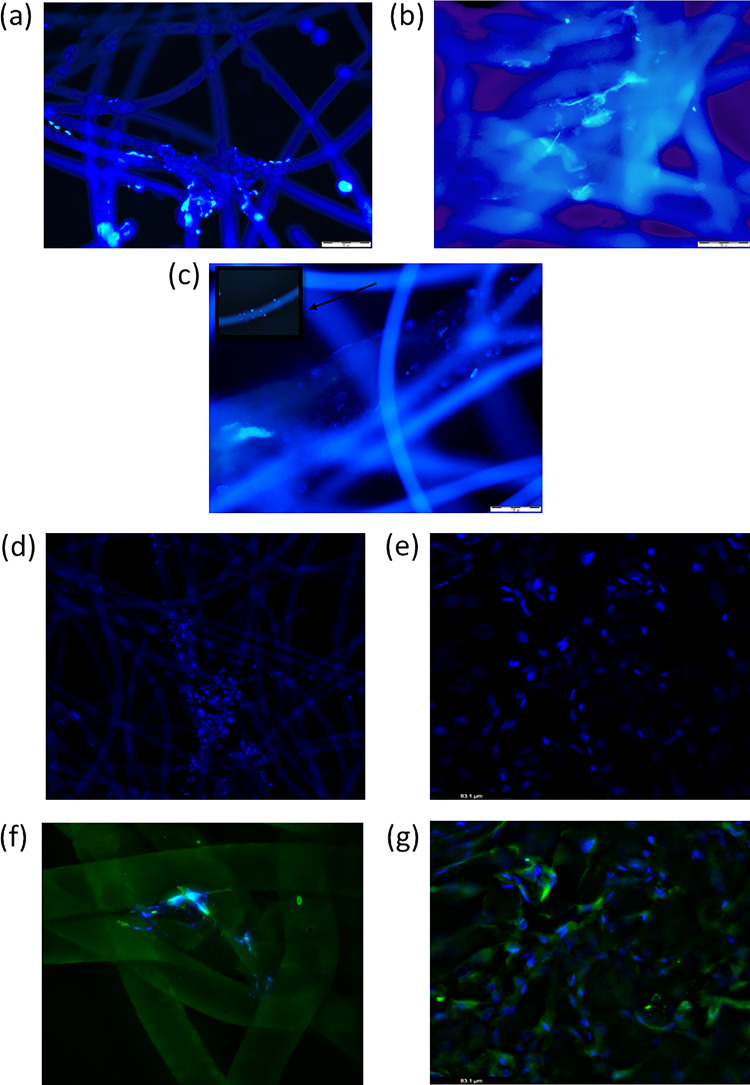
Representative cases of cells localization on Hyaff-11 membrane. (a) Chondrocytes growing on 3D structure. Chondrocytes form groups at the intersection and on single fibers (scale bar = 50 µm); (b) chondroinduced MSCs after 17 days. The connections between cells are visible (scale bar = 50 µm); (c) MSCs/chondrocytes after 17 days of culture in direct 3D co-culture model. The cells are mainly colonized at the intersection of fibers. The arrow indicate single fiber opsonized by cells (scale bar = 100 µm); (d) Localization of chondroinduced MSCs (magnification ×400); (e) Distribution of MSCs/chondrocytes after 17 days of culture in direct 3D co-culture model on single fibers (scale bar = 83.1 µm); (f, g) Localization and distribution of collagen in MSCs/chondrocytes after 17 days of culture in direct 3D co-culture model (magnification ×630; scale bar = 83.1 µm). Cells labelled with DAPI. Microphotographs (d–g) made by using the THUNDER Imaging Systems.

Firstly, the phenotype of cells differentiated in 3D co-culture system for 14 and 17 days were analyzed directly on fibers of the membrane (Fig. 2). The cells showed higher expression of type II collagen after 17 days of cultivation +/++ compared with 14 days +. No differences in aggrecan expression were observed at both time points (Table 3), whereas expression of type I and X collagen decreased from +++ at 14 days to +/++ at 17 days. In next step, cells were removed from the scaffold and expression of analyzed proteins was performed on cells samples (Table 2). The MSCs and chondrocytes in direct co-culture on Hyaff-11 at 17th day showed that type I and X collagen expression decreased compared with the 14th day of culture (*P *=* *0.0000; *P *=* *0.0003, respectively) (Table 2). The presence of type II collagen increased from 10 at 14th day to 80% of positive cells at 17th day of culture (*P *=* *0.0000). The number of positive cells with aggrecan achieved about 60% for both time points (*P *>* *0.05) (Table 2). The collagen was synthesized by cells growing on 3D membrane in both models (Fig. 3f and g). However, the higher level of collagens was found in direct co-culture model (Fig. 3g).

### Comparison of analyzed biomarkers between chondroinduced MSCs and MSCs/chondrocytes in direct co-culture on 3D membrane

The analyzed biomarkers were compered between chondroinduced MSCs and MSCs/chondrocytes in direct co-culture on Hyaff-11 in relation to time points. Significant differences were observed in type II, X collagen, aggrecan and CD105 expression in chondroinduced MSCs at 14 days of culture compered to co-culture at both time points (*P *≤* *0.05), as well as type I collagen, CD44 in chondroinduced MSCs after 14 days of culture versus co-culture at 17th day (*P *=* *0.0000). Chondroinduced MSCs after 17 days of differentiation showed significant differences in expression of type I collagen, aggrecan and CD105 versus direct co-culture at both time points. While significant differences in type II, X collagen, CD44 and CD90 expression was observed in chondroinduced MSCs at 17th day versus direct co-culture (*P *=* *0.0000 at 14th day; *P *=* *0.00003 at 17th day; *P *=* *0.0000 at 14th day; *P *=* *0.0000 at 14th day, respectively). The comparison was also made between chondrocytes and both groups in relation to both time points. Differences in aggrecan expression in chondrocytes were observed versus chondroinduced MSCs (*P *=* *0.02; *P *=* *0.01), versus co-culture (*P *=* *0.01; *P *=* *0.003) at both time points. The expression of type II collagen in chondrocytes showed differences compared with chondroinduced MSCs (*P *=* *0.000002 at 14th day) and co-culture (*P *=* *0.0000; *P *=* *0.03 at both time points) as well as type I collagen expression in chondrocytes showed significant differences compared with chondroinduced MSCs (*P *=* *0.00000) and co-culture (*P *=* *0.00000; *P *=* *0.03) at both time points. There were differences between presence of type X collagen in chondrocytes versus chondroinduced MSCs (*P *=* *0.00000; *P *=* *0.001 at both time points) and versus co-culture (*P *=* *0.007 at 14th day).

### Comparison of analyzed biomarkers in differentiated cells in 2D and 3D culture models

Comparison of analyzed biomarkers expression in chondroinduced MSCs in 2D and 3D standard model at two time points revealed significant differences for all analyzed markers (*P *≤* *0.05). Similar differences were observed between 2D and 3D co-culture system model for CD90, type I, II collagen, and aggrecan expression between analyzed groups at both time points (*P *≤* *0.05). Type X collagen, CD105 showed significant difference at 14th day of culture between groups (*P *=* *0.0000), while 17th day of direct 2D co-culture showed significant results compared with 17th day of 3D culture for type X collagen (*P *=* *0.003); versus 17th day for type I collagen (*P *=* *0.0000); versus both time points for CD44 (*P *=* *0.0000; *P *=* *0.02); versus 14th day for CD105 (*P *=* *0.0000). Figure 1A presents immunohistochemical staining where are visible differences between protein occurrence in cells after 17 days of direct co-culture in 2D and 3D model. It is observed increasing expression of immunopositive cells with type II collagen and aggrecan and decreasing expression of type I collagen on membrane.

The morphological features of cells in all culture models were analyzed. It was observed no clearly differences in cells morphology taking into account morphological features of physiological MSCs and chondrocytes between culture models (Fig. 1B and C). As shown in Fig. 1D and E the chondroinduced MSCs and MSCs/chondrocytes in direct co-culture revealed no pathological changes in their morphology. In this study cytoskeletal component like vimentin was also performed. The vimentin expression was present in higher number of cells in co-culture than in monoculture (Fig. 1F and G).

### Viability of cells on 3D structure

The MTT assay showed that the used membrane (Hyaff-11) is not toxic for the used cells. The viability of cells was 100% compared with control cells (Fig. 1H). The trypan blue staining confirmed MTT assay that on the scaffold most of the cells are alive.

### Localization of cultured cells on 3D structure

Chondrocytes and chondroinduced MSCs growing on 3D structure showed similar distribution on the scaffold surface. The growing cells revealed adhesion to the scaffold fibers, but mainly colonized between fibers (Fig. 3a and b). It was found that chondrocytes create connections between fibers. Such features were not observed in differentiated MSCs. There are no clearly differences between chondrocytes and differentiated MSCs location on used membrane.

The localization and distribution of cells growing in 3D co-culture was comparable with standard 3D culture. However, the density of growing cells on the surface and deeper layer was higher than normal 3D culture. The cells, similarly to chondrocytes in monoculture, create connections between fibers and are mainly colonized at the intersection of them. The cells also created a strong attachment to single fibers of Hyaff-11 (Fig. 3c). Using the THUNDER Imaging Systems the localization of differentiated cells growing on 3D structure was clearly visible (Fig. 3d and e). The cells opsonized mostly the fibers edges (Fig. 3e).

## Discussion

The articular cartilage is very important in smooth motion; however, the mechanical defects and osteoarthritis lead to cartilage tissue damage, which is why researchers are still looking for the best solutions to regenerate damaged tissue in order to improve the lives of patients with osteoarthritis and mechanical damage of the joints.

In our study, we analyzed changes of biological features of MSCs differentiated into chondrocytes and their location on 3D structure cultured in different conditions. Results from this study confirmed that chondrocytes cultured *in vitro* for the treatment of cartilage defects have limitations [8, 21, 26]. First of all, chondrocytes lose their chondrogenic phenotype during long-term *in vitro* culturing, thus its function disappears [8, 21, 26]. Similarly to Meretoja *et al*. [21], we showed that the presence of type II collagen decreases with each subsequent passage, while the presence of type I collagen increases. Also, clinical data revealed that the use of autologous chondrocyte implantation is not sufficient to proper cartilage treatment [27–29]. Therefore, in cartilage regeneration membranes are sought that will reflect the natural environment such as in the tissue and enhance the chondrogenic phenotype of chondrocytes. Increasing level of type II collagen expression and downregulation of type I collagen on Hyaff-11 compared with 2D culture observed in our study is in agreement with the study conducted by Grigolo *et al*. [14]. Our observation may be supported by earlier data, which showed that the hyaluronic acid as natural component of Hyaff-11 may enhance the regeneration of the damaged tissue by stimulating chondrocytes growth. On the other hand, the possibility of visualizing chondrocytes in 3D allowed us to describe their localization on the surface and in deeper layers of this membrane. We found a high concentration of cells on the intersection of fibers; however, the cells are strongly attached to single fibers. We may suggest that connections and interactions between cells observed in 3D membrane may have an impact on specific protein expression of chondrocytes. It seems reasonable to assume that chondrocytes growing on 3D have a more suitable microenvironment than in 2D [14].

The second approach in cartilage regeneration is based on MSCs culture. It has been reported that autologous bone marrow MSCs can promote tissue repair, which is why currently they are widely used for cartilage repair [8, 26]. We found that the MSCs line differentiation toward chondrocytes for 14 and 17 days in 2D culture revealed decreased expression of stem cells markers, type I collagen and lack of type II collagen and aggrecan expression, whereas MSCs differentiated on Hyaff-11 membrane showed higher expression of type II collagen, aggrecan and stem cells markers compered to 2D culture. This data is partly in agreement with published reports which showed that the potential of MSCs differentiating into chondrocytes may depend on culture conditions [30, 31].

Taking to account the limitations concerning MSCs differentiation towards chondrocytes, the new approach in cell-based therapies in cartilage tissue base on co-culture models was considered [27, 30]. This allows us to use a lower number of chondrocytes and MSCs than in monoculture. The data shows that direct contact of differentiated and undifferentiated cell populations in a co-culture influences synergistically the redifferentiation of chondrocytes and increases the chondrogenic differentiation of MSCs [32]. In the current study, direct co-culture of MSCs with chondrocytes resulted in increased chondrocytes biomarkers expression and chondrogenic differentiation of the MSCs compered to MSCs differentiation in standard culture. These results are comparable with previous data, which revealed an increased level of aggrecan [32, 33]. According to earlier reports, we may consider that the trophic factors produced by MSCs enhance the proliferation of chondrocytes and maintain their phenotype, whereas the specific factors produced by chondrocytes influence MSCs differentiation [28, 34].

Despite efforts to avoid dedifferentiation, current expansion methods still do not maintain the chondrocyte phenotype, so 3D co-culture systems may be a promising method, but the data concerning 3D co-culture MSCs with chondrocytes is limited [1]. However, there is no data showing direct co-culture of MSCs with chondrocytes on Hyaff-11 membrane. Interestingly, in the present study, after 17 days we observed increasing number of positive cells with type II collagen and aggrecan and decreasing type I collagen expression in 3D co-culture than standard 3D culture. This data indicates that this construction induces proper condition for MSCs differentiation and enhances chondrocytes redifferentiation. Our data is comparable with that of a study performed using dental pulp stem cells [35, 36]. According to previous studies our results revealed high level of type X collagen in chondroinduced MSCs and in co-culture cells at both time points in 2D and 3D model. This could be due to the fact that the cells were isolated from cartilage of patients with osteoarthritis not from healthy donors and at the beginning of the experiments it was 20% of cells with this protein. The data shows that chondrocytes obtained from unaffected cartilage distal to the damaged zone express also type X collagen like from damaged area [37]. Our results are concordant with Dehne *et al*. [38], who demonstrated increasing level of type X collagen in Hyaff-11 cultures. This increase of hypertrophic marker may be also caused by hyaluronic acid, what was found by Amann *et al*. [39]. Moreover, our results are partly comparable with Mardani *et al*. [40], who observed that during adipose derived stem cells differentiation toward chondrocytes shows increasing level of type X collagen during the culture time up to 21st day. Authors suggest that prolonged time of adipose-derived stem cells differentiation may decrease the expression of this protein. Nevertheless, there is significant increase of type II collagen, aggrecan and decreasing presence of type I collagen compered to monoculture what may indicate that co-culture on Hyaff-11 prevents from fibrocartilage formation. It was confirmed with no changes in cells morphology in comparison to chondrocytes. This may also lead to the conclusion that articular cartilage extracellular matrix may be formed. In the current study, we found that in 3D co-culture we observed a higher concentration of cells not only on the surface of the scaffold, but also in its deeper layer compared with standard 3D culture. We may conclude that culture conditions are an essential factor for chondrogenic potential. This observation may be supported by other data which revealed that microenvironmental factors in a co-culture system may enhance the cooperation between different subpopulations of cells, and the interactions between them are important for their growth and differentiation [1, 11, 21, 35, 36, 41].

In conclusion our findings suggest that, 3D co-culture induced suitable conditions for MSCs differentiation toward chondrocytes than 3D standard culture. Results from this study indicate that high expression of type II collagen and aggrecan in cells cultured in direct

3D co-culture model indicate that cooperation between different subpopulations may have a synergistic impact on MSCs chondrogenic potential. Revealed the high concentration and localization of cells growing in deeper layers of membrane in 3D co-culture indicate, that induced microenvironmental enhance cell migration within scaffold. Additionally, we suggest that co-culture model might be useful for construction a bioactive structure for cartilage tissue regeneration.

## References

[1] Cho H, Kim D, Kim K. Engineered co-culture strategies using stem cells for facilitated chondrogenic differentiation and cartilage repair. Biotechnol Bioproc E 2018;23:261–70.

[2] Davies R, Kuiper N. Regenerative medicine: a review of the evolution of autologous chondrocyte implantation (ACI) therapy. Bioengineering 2019;6:22. 10.3390/bioengineering6010022 PMC6466051 30871236

[3] de l’Escalopier N, Anract P, Biau D. Surgical treatments for osteoarthritis. Ann Phys Rehabil Med 2016;59:227–33. 27185463 10.1016/j.rehab.2016.04.003

[4] Brittberg M, Lindahl A, Nilsson A et al Treatment of deep cartilage defects in the knee with autologous chondrocyte transplantation. N Engl J Med 1994;331:889–95. 8078550 10.1056/NEJM199410063311401

[5] Rutgers M, Saris DB, Vonk LA et al Effect of collagen type I or type II on chondrogenesis by cultured human articular chondrocytes. Tissue Eng Part A 2013;19:59–65. 22861168 10.1089/ten.TEA.2011.0416

[6] Huang S, Song X, Li T et al Pellet coculture of osteoarthritic chondrocytes and infrapatellar fat pad-derived mesenchymal stem cells with chitosan/hyaluronic acid nanoparticles promotes chondrogenic differentiation. Stem Cell Res Ther 2017;8:264. 29141683 10.1186/s13287-017-0719-7 PMC5688648

[7] Yamagata K, Nakayamada S, Tanaka Y. Use of mesenchymal stem cells seeded on the scaffold in articular cartilage repair. Inflamm Regen 2018;38:4. 29560045 10.1186/s41232-018-0061-1 PMC5846298

[8] Huang H, Xu H, Zhang J. Current tissue engineering approaches for cartilage regeneration. In: *Cartilage Tissue Engineering and Regeneration Techniques* London: IntechOpen, 2019, 13. doi: 10.5772/intechopen.84429.

[9] Mekhileri NV, Lim KS, Brown GCJ et al Automated 3D bioassembly of micro-tissues for biofabrication of hybrid tissue engineered constructs. Biofabrication 2018;10:024103. 29199637 10.1088/1758-5090/aa9ef1

[10] Flanigan DC, Everhart JS, Early NA. Autologous chondrocyte implantation: scaffold-based solutions. In: *Cartilage Repair and Regeneration* London: IntechOpen, 2018, 13. doi: 10.5772/intechopen.70276.

[11] Lammi M, Piltti J, Prittinen J et al Challenges in fabrication of tissue-engineered cartilage with correct cellular colonization and extracellular matrix assembly. IJMS 2018;19:2700. 10.3390/ijms19092700 PMC6164936 30208585

[12] Benea H, Tomoaia G, Soritau O et al A review on the reconstruction of articular cartilage using collagen scaffolds. Rom Biotechnol Lett 2016;21:11735–43.

[13] Gao Y, Liu S, Huang J et al The ECM-cell interaction of cartilage extracellular matrix on chondrocytes. 2014. doi: 10.1155/2014/648459. PMC4052144 24959581

[14] Grigolo B, Lisignoli G, Piacentini A et al Evidence for redifferentiation of human chondrocytes grown on a hyaluronan-based biomaterial (HYAff 11): molecular, immunohistochemical and ultrastructural analysis. Biomaterials 2002;23:1187–95. 11791922 10.1016/s0142-9612(01)00236-8

[15] Brun P, Dickinson SC, Zavan B et al Characteristics of repair tissue in second-look and third-look biopsies from patients treated with engineered cartilage: relationship to symptomatology and time after implantation. Arthritis Res Ther 2008;10:R132. 19014452 10.1186/ar2549 PMC2656234

[16] Hollander AP, Dickinson SC, Sims TJ et al Maturation of tissue engineered cartilage implanted in injured and osteoarthritic human knees. Tissue Eng 2006;12:1787–98. 16889509 10.1089/ten.2006.12.1787

[17] Cavallo C, Desando G, Columbaro M et al Chondrogenic differentiation of bone marrow concentrate grown onto a hylauronan scaffold: rationale for its use in the treatment of cartilage lesions. J Biomed Mater Res Part Res 2013;101A:1559–70. 10.1002/jbm.a.34460 23135955

[18] Ullah I, Subbarao RB, Rho GJ. Human mesenchymal stem cells - current trends and future prospective. Biosci Rep 2015;35:1–18. 10.1042/BSR20150025 PMC4413017 25797907

[19] Levorson EJ, Santoro M, Kasper FK et al Direct and indirect co-culture of chondrocytes and mesenchymal stem cells for the generation of polymer/extracellular matrix hybrid constructs. Acta Biomater 2014;10:1824–35. 24365703 10.1016/j.actbio.2013.12.026 PMC3976699

[20] Cooke ME, Allon AA, Cheng T et al Structured three-dimensional co-culture of mesenchymal stem cells with chondrocytes promotes chondrogenic differentiation without hypertrophy. Osteoarthr Cartil 2011;19:1210–8. 10.1016/j.joca.2011.07.005 PMC3188316 21816228

[21] Meretoja VV, Dahlin RL, Wright S et al Articular chondrocyte redifferentiation in 3D co-cultures with mesenchymal stem cells. Tissue Eng Part C Methods 2014;20:514–23. 24387702 10.1089/ten.tec.2013.0532 PMC4025602

[22] Ma Q, Tian T, Liu N et al Application of Stem Cells and the Factors Influence Their Differentiation in Cartilage Tissue Engineering. Cham: Humana Press, 2017,1–20.

[23] Yang Y-H, Lee AJ, Barabino GA. Coculture-driven mesenchymal stem cell-differentiated articular chondrocyte-like cells support neocartilage development. Stem Cells Transl Med 2012;1:843–54. 23197696 10.5966/sctm.2012-0083 PMC3659664

[24] Hubka KM, Dahlin RL, Meretoja VV et al Enhancing chondrogenic phenotype for cartilage tissue engineering: monoculture and coculture of articular chondrocytes and mesenchymal stem cells. Tissue Eng Part B Rev 2014;20:641–54. 24834484 10.1089/ten.teb.2014.0034 PMC4241977

[25] Isyar M, Yilmaz I, Yasar Sirin D et al A practical way to prepare primer human chondrocyte culture. J Orthop 2016;13:162–7. 27408489 10.1016/j.jor.2016.03.008 PMC4919283

[26] De Bari C, Roelofs AJ. Stem cell-based therapeutic strategies for cartilage defects and osteoarthritis. Curr Opin Pharmacol 2018;40:74–80. 29625333 10.1016/j.coph.2018.03.009

[27] Armiento AR, Stoddart MJ, Alini M et al Biomaterials for articular cartilage tissue engineering: learning from biology. Acta Biomater 2018;65:1–20. 29128537 10.1016/j.actbio.2017.11.021

[28] Liu Y, Zhou G, Cao Y. Recent progress in cartilage tissue engineering—our experience and future directions. Engineering 2017;3:28–35.

[29] Madeira C, Santhagunam A, Salgueiro JB et al Advanced cell therapies for articular cartilage regeneration. Trends Biotechnol 2015;33:35–42. 25466849 10.1016/j.tibtech.2014.11.003

[30] Dahlin RL, Kinard LA, Lam J et al Articular chondrocytes and mesenchymal stem cells seeded on biodegradable scaffolds for the repair of cartilage in a rat osteochondral defect model. Biomaterials 2014;35:7460–9. 24927682 10.1016/j.biomaterials.2014.05.055 PMC4109803

[31] Somoza RA, Welter JF, Correa D et al Chondrogenic differentiation of mesenchymal stem cells: challenges and unfulfilled expectations. Tissue Eng Part B Rev 2014;20:596–608. 24749845 10.1089/ten.teb.2013.0771 PMC4241862

[32] Kim TW, Lee MC, Bae HC et al Direct coculture of human chondrocytes and synovium-derived stem cells enhances in vitro chondrogenesis. Cell J 2018;20:53–60. 29308619 10.22074/cellj.2018.5025 PMC5759681

[33] Zuo Q, Cui W, Liu F et al Co-cultivated mesenchymal stem cells support chondrocytic differentiation of articular chondrocytes. Int Orthopaed 2013;37:747–52. 10.1007/s00264-013-1782-z PMC3609966 23354690

[34] Matsiko A, Levingstone T, O'Brien F. Advanced strategies for articular cartilage defect repair. Materials 2013;6:637–68. 28809332 10.3390/ma6020637 PMC5452095

[35] Rizk A, Rabie A. Human dental pulp stem cells expressing transforming growth factor β3 transgene for cartilage-like tissue engineering. Cytotherapy 2013;15:712–25. 23474328 10.1016/j.jcyt.2013.01.012

[36] Mata M, Milian L, Oliver M et al *In vivo* articular cartilage regeneration using human dental pulp stem cells cultured in an alginate scaffold: A preliminary study. Stem Cells Int 2017;2017:1–9. 10.1155/2017/8309256 PMC5603743 28951745

[37] Yang KGA, Saris DBF, Geuze RE et al Altered in vitro chondrogenic properties of chondrocytes harvested from unaffected cartilage in osteoarthritic joints. Osteoarthr Cartil 2006;14:561–70. 10.1016/j.joca.2005.12.002 16735197

[38] Dehne T, Karlsson C, Ringe J et al Chondrogenic differentiation potential of osteoarthritic chondrocytes and their possible use in matrix-associated autologous chondrocyte transplantation. Arthritis Res Ther 2009;11:R133. 19723327 10.1186/ar2800 PMC2787268

[39] Amann E, Wolff P, Breel E et al Hyaluronic acid facilitates chondrogenesis and matrix deposition of human adipose derived mesenchymal stem cells and human chondrocytes co-cultures. Acta Biomater 2017;52:130–44. 28131943 10.1016/j.actbio.2017.01.064

[40] Mardani M, Hashemibeni B, Masoud Ansar M et al Comparison between chondrogenic markers of differentiated chondrocytes from adipose derived stem cells and articular chondrocytes in vitro. Iran J Basic Med Sci. 2013;16:763–73. 23997902 PMC3758031

[41] Ishikawa S, Iijima K, Sasaki K et al Cartilage differentiation of bone marrow-derived mesenchymal stem cells in three-dimensional silica nonwoven fabrics. Appl Sci 2018;8:1398. 10.1021/acsomega.8b01139 PMC6645240 31459146

